# Titin Truncating Variants in Dilated Cardiomyopathy – Prevalence and Genotype-Phenotype Correlations

**DOI:** 10.1371/journal.pone.0169007

**Published:** 2017-01-03

**Authors:** Maria Franaszczyk, Przemyslaw Chmielewski, Grazyna Truszkowska, Piotr Stawinski, Ewa Michalak, Malgorzata Rydzanicz, Malgorzata Sobieszczanska-Malek, Agnieszka Pollak, Justyna Szczygieł, Joanna Kosinska, Adam Parulski, Tomasz Stoklosa, Agnieszka Tarnowska, Marcin M. Machnicki, Bogna Foss-Nieradko, Malgorzata Szperl, Agnieszka Sioma, Mariusz Kusmierczyk, Jacek Grzybowski, Tomasz Zielinski, Rafal Ploski, Zofia T. Bilinska

**Affiliations:** 1 Department of Medical Biology, Molecular Biology Laboratory, Institute of Cardiology, Warsaw, Poland; 2 Unit for Screening Studies in Inherited Cardiovascular Diseases, Institute of Cardiology, Warsaw, Poland; 3 Department of Genetics, Institute of Physiology and Pathology of Hearing, Kajetany/Warsaw, Poland; 4 Department of Medical; Genetics, Medical University of Warsaw, Warsaw, Poland; 5 Department of Heart Failure and Transplantology, Institute of Cardiology, Warsaw, Poland; 6 Department of Cardiomyopathies, Institute of Cardiology, Warsaw, Poland; 7 Department of Cardiac Surgery and Transplantology, Institute of Cardiology, Warsaw, Poland; 8 Department of Immunology, Center for Biostructure Research, Medical University of Warsaw, Warsaw, Poland; 9 Postgraduate School of Molecular Medicine, Medical University of Warsaw, Warsaw, Poland; University of Texas MD Anderson Cancer Center, UNITED STATES

## Abstract

*TTN* gene truncating variants are common in dilated cardiomyopathy (DCM), although data on their clinical significance is still limited. We sought to examine the frequency of truncating variants in *TTN* in patients with DCM, including familial DCM (FDCM), and to look for genotype-phenotype correlations. Clinical cardiovascular data, family histories and blood samples were collected from 72 DCM probands, mean age of 34 years, 45.8% FDCM. DNA samples were examined by next generation sequencing (NGS) with a focus on the *TTN* gene. Truncating mutations were followed up by segregation study among family members. We identified 16 *TTN* truncating variants (*TTN* trunc) in 17 probands (23.6% of all cases, 30.3% of FDCM, 17.9% of sporadic DCM). During mean 63 months from diagnosis, there was no difference in adverse cardiac events between probands with and without TTN truncating mutations. Among relatives 29 mutation carriers were identified, nine were definitely affected (31%), eight probably affected (27.6%) one possibly affected (3.4%) and eleven were not affected (37.9%). When relatives with all affected statuses were combined, disease penetrance was still incomplete (62.1%) even after exclusion of unaffected relatives under 40 (82%) and was higher in males versus females. In all mutation carriers, during follow-up, 17.4% had major adverse cardiac events, and prognosis was significantly worse in men than in women. In conclusion, *TTN* truncating variants were observed in nearly one fourth of young DCM patient population, in vast majority without conduction system disease. Incomplete penetrance suggests possible influence of other genetic and/or environmental factors on the course of cardiotitinopathy. Counseling should take into account sex and incomplete penetrance.

## Introduction

Whereas mutations in numerous loci have been known to predispose to dilated cardiomyopathy (DCM) [1], recently titin gene (*TTN*) emerged as a major DCM locus with truncating mutations found in one fifth to one fourth of patients, especially those with familial forms of the disease [2–4]. However, some doubt about pathogenicity of *TTN* truncating variants was raised by their presence in 1% of hypertrophic cardiomyopathy (HCM) patients and in 3% of controls [2], and by their co-occurrence with additional known disease-causing variants in the patients [5]. Recently, an exhaustive analysis of *TTN* truncated transcripts expression in the heart muscle was published showing that unlike in controls, *TTN* truncating mutations in DCM patients were predominantly located in the sarcomeric A-band region of the protein, occurred closer to the protein’s carboxyl terminus and were present in exons that were abundantly transcribed in the heart muscle [6].

Our aims were: (1) to identify genetic background in a cohort of DCM patients referred for clinical genetic diagnosis with a focus on *TTN* truncating mutations, and (2): to examine genotype-phenotype correlations.

## Materials and Methods

### Patient population

All patients and relatives signed written informed consent in accordance with the Declaration of Helsinki. The study was approved by local Bioethics Committee of Institute of Cardiology with approval number 1276.

The study cohort was drawn from all index patients referred for clinical genetic testing to Unit for Screening Studies in Inherited Cardiovascular Diseases with the diagnosis of DCM from 2012 to 2014. The cohort comprised of 72 unrelated DCM probands, all were Caucasian, 48 were male (66.7%). In addition, we performed segregation study among available 44 relatives of *TTN* truncating mutation positive probands. DCM was defined according to ESC (European Society of Cardiology) criteria, with left ventricular ejection fraction below 45% and left ventricular end-diastolic diameter >117% of the predicted value corrected for age and body surface area [7]. In all probands, coronary angiography, or more recently coronary computed tomography angiography was performed. Data concerning the heart transplant recipients were reviewed to confirm the diagnosis of DCM prior to heart transplantation. The disease was considered as familial when two subjects in the family met the same diagnostic criteria for DCM as proband. Diagnostic criteria in relatives were defined based on position statement of the ESC Working Group on Myocardial and Pericardial Diseases published by Pinto et al [8], with definite disease in relatives who met criteria for DCM. Definitely affected status was only assigned when systolic dysfunction was present with LVEF≤50% and LVEDD>117% in patients who had no other known causes (including coronary artery disease (CAD)) leading to systolic dysfunction. Probable disease was diagnosed in the relatives, carriers of causative mutation in the proband, whenever one major criterion that is unexplained decrease of LVEF ≤50% but >45%, or unexplained LVED dilatation was present while possible disease, in the same population, was diagnosed when one minor criterion was present, e.g. unexplained ventricular arrhythmia (>100 ventricular premature beats per hour in 24h monitoring or non-sustained ventricular tachycardia ≥3 beats at a rate ≥120 beats per minute). However, similarly as in probands, we used the method of Henry [9] in relation to indexation of left ventricular enddiastolic dimension as widely published and easy to make comparisons with other publications. Medical records of relatives diagnosed in our center, who were hospitalized and/or died earlier, were analyzed to ascertain familial form of the disease. Genetic testing was offered to all probands and they all agreed to participate in the study. The informed and consenting relatives of the probands underwent a clinical examination, 12-lead electrocardiography, two-dimensional Doppler echocardiography, 24-hour Holter electrocardiography (ECG) monitoring, serum creatine phosphokinase (CPK) examination and blood sampling for genetic testing. Cardiac magnetic resonance study was performed based on clinical decision.

Mean age of probands at diagnosis was 33.8±13.6 years, and mean left ventricular ejection fraction at diagnosis was 24%. Of 72 probands, 33 (45.8%) were with familial DCM (FDCM), and 39 (54.2%) with sporadic DCM.

### DNA sequencing and *TTN* mutation analysis

DNA was extracted from the peripheral blood by phenol extraction. Genetic testing in probands was performed using next generation sequencing (NGS). 44 patients were analyzed by whole exome sequencing (WES), in 22 TruSight One (TSO) sequencing panel and in 6 sequencing of a custom panel of 35 genes involved in cardiomyopathies (*ABCC9*, *ACTC1*, *ACTN2*, *ANKRD1*, *BAG3*, *CRYAB*, *CSRP3*, *DES*, *EMD*, *ILK*, *LAMA4*, *LDB3*, *LMNA*, *MYBPC3*, *MYH6*, *MYH7*, *MYL2*, *MYL3*, *MYPN*, *PDLIM3*, *PLN*, *PSEN1*, *PSEN2*, *RBM20*, *SCN5A*, *SGCD*, *TAZ*, *TCAP*, *TMPO*, *TNNC1*, *TNNI3*, *TNNT2*, *TPM1*, *TTN*, *VCL*) were performed. The detailed NGS approach for each proband with *TTN* truncating variant found is given in S1 Table. WES libraries were constructed using TruSeq Exome Enrichment Kit (Illumina) or Nextera Rapid Capture Exome Kit (Illumina) as described previously [10]. Except for the different set of enrichment probes TSO sequencing was performed similarly to WES. Targeted sequencing of the 35 genes was performed using a custom design SeqCap EZ Choice Library (Roche NimbleGen) of ~0.17 Mb target genomic sequences. Whole procedure was carried out according to SeqCap EZ Library SR User’s Guide v.3.0. All libraries were pair-end sequenced on Illumina HiSeq 1500 with minimum depth of 10 reads (ge10) for at least 80% of respective NGS target regions and for 88.4% for the entire coding region of *TTN* defined as *TTN* N2BA transcript NM_001256850.1. Mean ge10 was 97.5±1.99 for N2BA *TTN* transcript. The mean coverage was 61.5±43.1 for NGS target regions and 66.6±41.0 for N2BA transcript NM_001256850.1. The NGS parameters are given for N2BA transcript as we considered only variants in cardiac *TTN* transcript NM_001256850.1.

*TTN* truncating variants identified with NGS were followed-up in probands and relatives with Sanger sequencing using a 3500xL Genetic Analyzer (Life Technologies, Carlsbad, CA, USA) and BigDye Terminator v3.1 Cycle Sequencing Kit (Life Technologies) according to the manufacturer’s instructions. The results were analyzed with Variant Reporter 1.1 Software (Life Technologies). List of primers specific to each *TTN* truncating variant is available in S2 Table. The frequencies of variants were compared to genomic databases: Phase 3 of 1000 Genomes (http://www.1000genomes.org/), NHLBI GO Exome Sequencing Project (ESP) 6500 (https://esp.gs.washington.edu/drupal/) and Version 0.3 of ExAC (http://exac.broadinstitute.org/).

### Statistical analysis

All results for categorical variables were presented as numbers and percentages and for continuous variables as mean and standard deviation (SD) or median. The Fisher’s exact test was used for comparison of categorical variables. All tests were two-sided with the significance level of p<0.05. Kaplan-Meier curves were compared with log-rank test. Statistical analyses were performed with statistical package STATISTICA v6.

## Results

### Molecular findings in the DCM cohort

**Fig 1:**
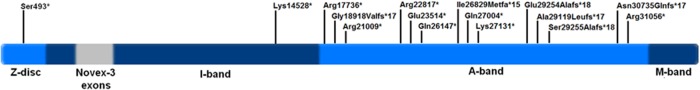
The distribution of *TTN* truncating variants found in this study. Bands/regions of *TTN* gene are shown as boxes.

We identified 16 different *TTN* truncating variants (*TTN* trunc, one identified twice) in 17 (23.6%) of 72 subjects (Table 1). Ten of 33 FDCM probands (30.3%) carried a *TTN* truncating variant (*TTN* trunc) compared to seven of 39 sporadic DCM cases (17.9%). There were ten nonsense and six frameshift mutations but no splice variants. Of the 16 variants, 3 were described before—two were found in large population study by Roberts et al. [11] (p.Arg31056* in end stage of DCM and p.Arg21009* in the UK prospective DCM cohort) and one (p.Ser28693Ilefs*2) in a family with adult-onset DCM [12]. Fourteen variants (87.5%) were localized in the A-band region, one (6.25%) was in the I-band, and one (6.25%) was located in the Z disc. (Fig 1, S3 Table). All of these *TTN* mutations were located in symmetric exons. The majority of probands (15/17, 88.2%) had mutations localized in the A-band. The only variant found outside A-band and adjacent region of I-band was found in a family where cosegregation was nearly full with only one of 6 family members, 18 years old woman, being unaffected (see S2 Fig, family DCM097).

**Table 1:** List of *TTN* truncating variants identified in the study group annotated to transcript NM_001267550.2.

Genomic position	TTN truncating variant	Family
**Frameshift deletions**		
chr2:179463684	p.Gly18918Valfs*17/c.56751_56752delAG	DCM023
chr2:179430371	p.Ile26829Metfs*15/c.80486delT	DCM033
chr2:179422725	p.Ala29119Leufs*17/c.87355delG	DCM082, DCM102
chr2:179422231	p.Ser29255Alafs*18/c.87757delA	DCM092
**Frameshift insertions**		
chr2:179424782	p.Ser28693Ilefs*2/c.86078insA	DCM081
chr2:179414153	p.Asn30734Glnfs*17/c.92200insC	DCM109
**Nonsense (stop) variants**		
chr2:179658189	p.Ser493*/c.1478C>A	DCM097
chr2:179497039	p.Lys14528*/c.43582A>T	DCM078
chr2:179472209	p.Arg17736*/c.53206C>T	DCM113
chr2:179453427	p.Arg21009*/c.63025C>T	DCM019
chr2:179442793	p.Arg22817*/c.68449C>T	DCM075
chr2:179440319	p.Glu23514*/c.70540G>T	DCM132
chr2:179432420	p.Gln26147*/c.78439C>T	DCM134
chr2:179429849	p.Gln27004*/c.81010C>T	DCM029
chr2:179429468	p.Lys27131*/c.81391A>T	DCM036
chr2:179413187	p.Arg31056*/c.93166C>T	DCM008

To verify the possibility of relatedness of two probands sharing the same *TTN* truncating variant we have used WES data and compared rare (<0.01 in databases cited above) variants in coding sequence excluding sex chromosomes. After such filtering among 376 variants in proband from DCM082 family and 223 variants in proband from DCM102 family the only one shared variant between these patients was *TTN* truncating variant which suggest no relatedness. This conclusion is further supported by internal comparisons between samples from related and unrelated subjects analyzed by WES in our lab for other purposes (i.e. 1–3 shared variants found for 4 unrelated samples out of 296–335 variants, 34 variants shared between two 4th degree relatives out of 252 and 276 variants in a similar analysis as described above).

In our group of 72 probands we also found 41 missense variants in cardiac transcript N2BA– 10 in 17 *TTN* trunc carriers (58.8% of carriers), 30 in non-carriers (54.5% of non-carriers) and 1 shared between two probands one with and the other without *TTN* truncating variant. Full list of missense *TTN* variants in our cohort is shown in S4 Table. While we did not test if they are located on the same or the other allele as *TTN* truncating variants similar prevalence among carriers vs. non-carriers argues against significant effect among the latter.

#### Clinical characteristics of DCM probands

Table 2 shows comparison of clinical data in *TTN* trunc positive (n = 17) versus *TTN* trunc negative DCM patients (n = 55). Conduction disease was defined as the presence of left bundle branch block (LBBB) and/or atrioventricular block (AVB). One *TTN* trunc positive proband had LBBB (5.9%) whereas among 55 *TTN* trunc negative probands 21 (38.2%) had conduction disease (13 had LBBB, 4 had LBBB and AVB, and 4 had AVB). However, the difference in prevalence of conduction disease between *TTN* trunc carriers and noncarriers was not significant after correction for number of comparisons (n = 21) (Table 2). There were no significant differences in age at diagnosis, sex, the presence of familial disease, symptoms and cardiac assessment between these groups.

**Table 2:** Comparison of clinical data on *TTN* trunc positive versus *TTN* trunc negative DCM probands.

	*TTN*(-) probands	*TTN*(+) probands	p
**N**	55 (76.4%)	17 (23.6%)	
**Age at diagnosis**	33.8±14.4	33.3±11.1	0.86
**Male sex n (%)**	65.5% (36)	70.6% (12)	0.69
**Familial form**	41.8% (23)	58.8% (10)	0.24
**Symptoms**			
**Acute onset heart failure**	30.9% (17)	23.5% (4)	0.76
**Palpitations**	10.9% (6)	0	0.32
**Decreased exercise tolerance**	49.1% (27)	76.5% (13)	0.26
**NYHA class at onset**	2.7±1.0	2.9±0.8	0.36
**Cardiac assessment**			
**LVEF % (mean±SD)**	24.4±10.2	24.5±9.0	0.69
**Sinus rhythm**	85.5% (47)	88.2% (15)	1.00
**AF/PAF**	30.9% (17)	35.3% (6)	0.73
**LBBB and/or AVB**	38.2% (21)	5.9% (1)	0.01^a^
**Outcome**			
**Time from diagnosis**	63.7±71.3	63.6±63.9	0.98
**Major adverse cardiac events**	30.9% (17)	29.4% (5)	0.91
**HF Death**	3.6% (2)	0.0%	1.00
**HTX**	27.3% (15)	23.5% (4)	1.00
**LVAD as bridge to recovery**	0	5.9% (1)	0.24
**Stable course**	27.3% (15)	17.7% (3)	0.53
**Improvement**	41.8% (23)	52.9% (9)	0.42
**PM**	9.1% (5)	11.8% (2)	0.67
**ICD**	50.9% (28)	41.2% (7)	0.58
**CRT-D**	23.6% (13)	5.9% (1)	0.16

^a^—p-value = 0.21 when corrected for multiple comparisons, correction factor—21.

NYHA—New York Heart Association; AF—permanent atrial fibrillation; PAF—paroxysmal atrial fibrillation; LBBB—left bundle branch block; nsVT—non-sustained ventricular tachycardia; LVEF—left ventricular ejection fraction; SD—standard deviation; DCM—dilated cardiomyopathy; HCM—hypertrophic cardiomyopathy; LVSD—left ventricular systolic dysfunction; LVNC—left ventricular non-compaction; SAE—serious adverse events; HF—heart failure; HTX—heart transplantation; LVAD—left ventricular assist device; LV—left ventricular; PM—pacemaker; ICD—implantable cardioverter defibrillator; CRT-D—cardiac resynchronisation therapy defibrillator.

**Fig 2:**
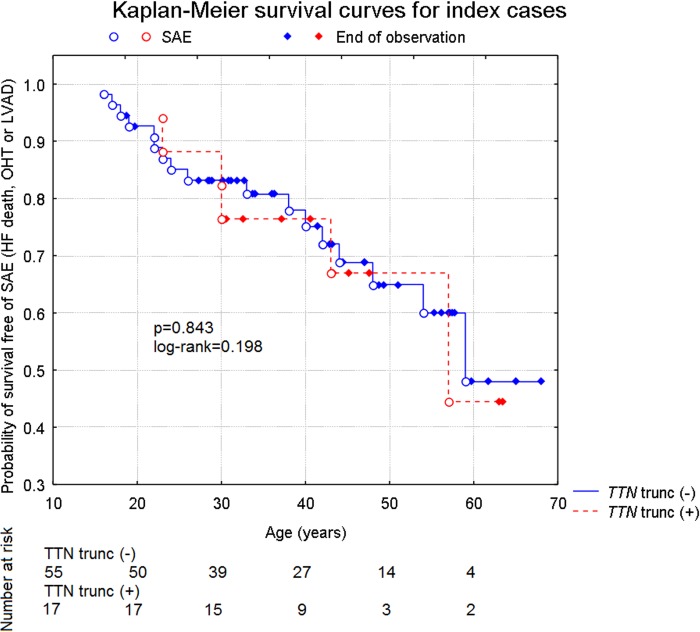
Kaplan-Meier cumulative survival curves for serious adverse events (SAE) (HF death, orthotopic heart transplant (OHT) or LVAD) in 72 patients with dilated cardiomyopathy, carriers of *TTN* truncating variants and non-carriers, p = 0.843, log-rank test = -0.198.

During mean 63 months from diagnosis there were 22 (30.5%) major adverse cardiac events in the whole group (n = 72). Kaplan-Meier survival curve did not show difference in outcome in *TTN* trunc carriers versus noncarriers (p = 0.843, log-rank test = -0.198, Fig 2).

#### Clinical characteristics of all *TTN* truncating mutations carriers

Evaluation of probands’ relatives identified 29 mutation carriers and 15 subjects without the mutation. Of the total of 29 relatives with *TTN* mutations 9 relatives (31%) had definitely affected status, 8 (27.6%) had probably affected status and one (3.4%) had possibly affected status (Table 3). The remaining 11 relatives (37.9%) were categorized as not affected. Of the 8 relatives with probably affected status 6 had left ventricular systolic dysfunction, and 2 had left ventricular dilatation. One female relative was categorized as possible affected with bursts of non-sustained ventricular tachycardia (nsVT) on 24-hour Holter electrocardiographic monitoring. Pedigrees for 46 mutation carriers from 17 families of *TTN* trunc positive DCM families are shown in S1 and S2 Figs. Detailed combined clinical and genetic data of the 46 *TTN* truncating mutation carriers are available on request from the authors.

**Table 3:** Clinical characteristics of affected and not affected *TTN* trunc positive relatives.

	All *TTN* (+) relatives	*TTN* (+) relatives affected def	*TTN* (+) relatives affected prob	*TTN* (+) relatives affected poss	*TTN* (+) relatives not affected
**N (%)**	29 (100)	9 (31)	8 (27.6)	1 (3.4)	11 (37.9)	
**Age at diagnosis**	38.9±16.6	41.8±17.7	42.3±19.0	39	34±15.1	
**Female sex n (%)**	16 (55.2)	4 (44.4)	2 (25)	1 (100)	9 (81.8)	
**Symptoms**						
**Acute onset heart failure**	2 (6.9)	2 (22.2)	0 (0)	0	0	
**Sudden cardiac arrest**	1 (3.4)	1 (11.1)	0 (0)	0	0	
**Decreased exercise tolerance**	5 (17.2)	5 (55.6)	0 (0)	0	0	
**NYHA class at onset**	3.0±0.9	3.0±0.9	-	-	-	
**Asymptomatic**	21 (72.4)	1 (11.1)	8 (100)	1(100)	11 (100)	
**Cardiac assessment**						
**LVEF % (mean±SD)**	47.7±16.0	28.1±12.1	49.9±2.9	55	61.4±5.9	
**LVEF<45%**	8 (27.6)	8 (88.9)	0	0	0	
**45%≤LVEF≤50%**	7 (24.1)	1 (11.1)	6 (75)	0	0	
**LVEDD>117%**	11 (37.9)	9 (100)	2 (25)	0	0	
**Sinus rhythm**	27 (27.6)	7 (77.8)	8 (100)	1 (100)	11 (100)	
**AF/PAF**	4 (13.8)	4 (44.4)	0	0	0	
**LBBB**	2 (6.9)	2 (6.9)	0	0	0	
**NsVT or >100 Vex/h**	5 (17.2)	3 (33.3)	1 (12.5)	1 (100)	0	
**Outcome**						
**Time from diagnosis (months)**	34.3±49.1	68±83.7	19±11.5	20	22.1±15.4	
**Major adverse cardiac events**	3 (10.3)	3 (33.3)	0	0	0	
**HF Death**	2 (6.9)	2 (22.2)	0	0	0	
**HTX**	1 (3.4)	1 (11.1)	0	0	0	

For Table legend see Table 2. affected def–affected-definite disease, affected prob–affected-probable disease, affected poss–affected-possible disease; nsVT—non-sustained ventricular tachycardia; Vex–ventricular extrasystole.

S5 Table shows combined data on all *TTN* trunc positive carriers with DCM (n = 26) and comparison of *TTN* trunc positive probands (n = 17) with *TTN* trunc positive DCM relatives (n = 9). There was no difference in the severity of the disease at diagnosis (mean LVEF 24.5±9.0% in probands and 28.1±12.1% in relatives, p = 0.49) and outcome between these two DCM patients’ subgroups, namely major adverse cardiac events were present in 5/17 probands and in 3/9 affected DCM relatives, p = NS.

#### Among *TTN* truncating mutation carriers disease penetrance and risk of major adverse cardiac events are higher in males than females

Overall disease penetrance among relatives carrying *TTN* truncating mutations was 62.1% and did not clearly increase with age being 60% by age 50 (n = 20), 56% by age 60 (n = 25) and 62% by age 70 (n = 29). After excluding all unaffected *TTN* trunc relatives younger than 40 years the penetrance was 82%. However, we noted that in *TTN* trunc relatives older than 59 years (n = 5, 3 females) the penetrance was 100%.

**Fig 3:**
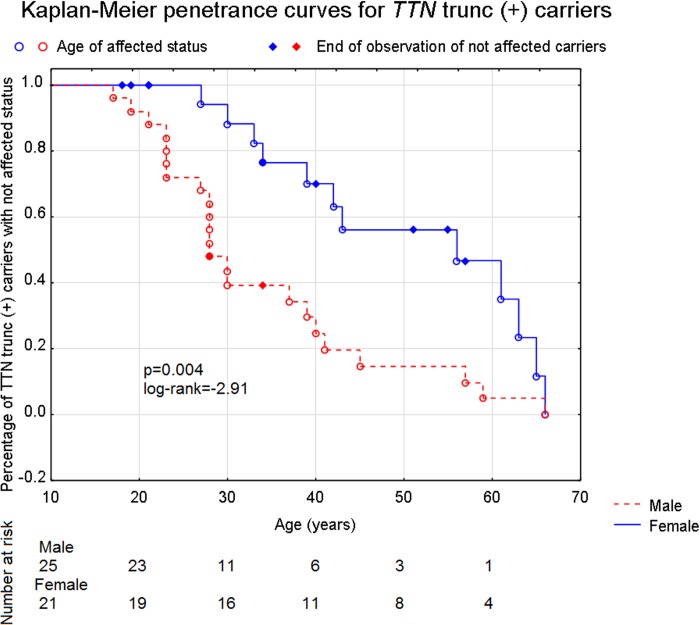
Kaplan-Meier curves showing the freedom from disease for *TTN* trunc carriers (n = 46) for female (n = 21) and male (n = 25), p = 0.004, log-rank test = -2.91.

We observed that the penetrance was sex dependent. Fig 3 presents Kaplan-Meier curves for freedom from combined affected statuses (age at diagnosis for affected and age at genetic inquest for not affected patients) in all *TTN* trunc carriers stratified by sex. Median age of onset in males was estimated as 28 years (range from 26 to 30) and in females as 56 years (range from 33 to 79) (p = 0.004, log-rank test = -2.91). Though we believe that there is no reason to exclude probands, as the criterion used in this analysis is age of onset, which is independent from patient’s proband/relative status, when the analysis was restricted to *TTN* trunc relatives the effect was still statistically significant (p = 0.029, log-rank test = -2.18). Furthermore, according to Kaplan-Meier analysis based on all *TTN* trunc individuals the estimated cumulative risk of DCM was 60.8% at the age of 33 and 95.1% at 61 years in males, and 17.6% and 53.9% in females, respectively.

**Fig 4:**
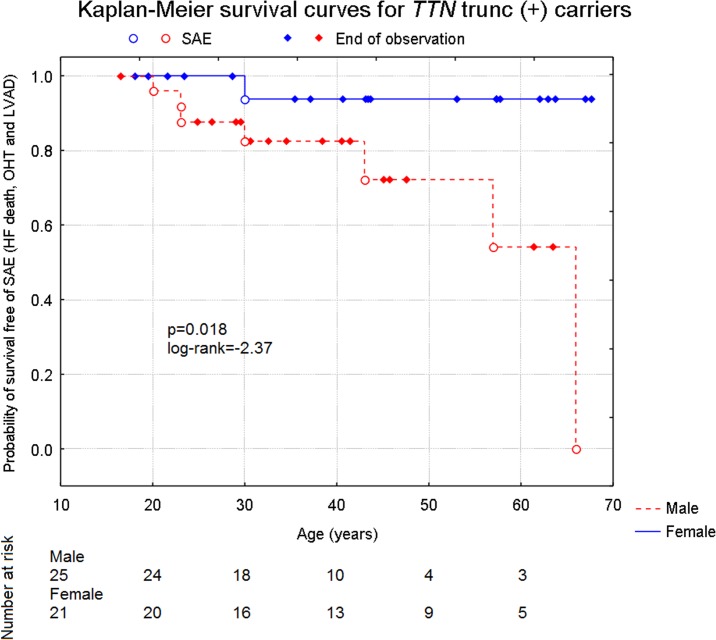
Kaplan-Meier cumulative survival curves for SAE (HF death, OHT or LVAD) in 46 carriers of *TTN* truncating variants, males and females, p = 0.018, log-rank test = -2.37.

The incidence of major adverse cardiac events among those with *TTN* mutations was also sex dependent. In all mutation carriers (n = 46), during 45.3±56.3 months of follow-up, 8 (17.4%) had major adverse cardiac events. Fig 4 shows Kaplan-Meier survival curve indicating worse prognosis in male carriers of *TTN* truncating variants in comparison to female carriers. The difference between these two groups was statistically significant (p = 0.018, log-rank test = -2.37).

#### Additional possible disease causing variants

In 8/17 (47%) of *TTN* trunc positive probands additional rare (frequency lower than 0.005 in 1000Genomes, ESP, and ExAC databases) variants in known cardiomyopathies’ genes were found, namely *ACTN2*, *DSP*, *LDB3*, *MYH6*, *MYH7*, *PKP2*, *SCN5A*, *TNNI3*. All of them were missense variants with variable bioinformatics predictions of pathogenicity and clinical significance (S6 Table). Two of them were described before: *DSP* variant as unclassified in proband with arrhythmogenic right ventricular cardiomyopathy [13] and *MYH7* variant as possibly pathogenic but coexisting with another possibly pathogenic variant in *LDB3* gene in proband with familial dilated cardiomyopathy [12]. The segregation of additional variants in studied families is included in S1 and S2 Figs.

Identification of additional variants in nearly half of probands carrying the *TTN* trunc raised a question about their possible influence on outcome. Among 9 affected relatives of these probands, 7 (78%) had also additional variants, there were only 2 affected *TTN* trunc positive relatives in whom additional variant was not present whereas none of the affected relatives carried only the additional variant. Taken together these data are consistent with the hypothesis that in these families DCM is caused by *TTN* trunc but the numbers are too small for definite conclusions.

## Discussion

While studying 72 DCM patients we identified 16 different *TTN* truncating variants, 13 of them novel. An important feature of our study is the examination of young DCM patient population with a mean age of 34 years.

The observed frequency of *TTN* truncating variants among our unselected young DCM patient population (23.6%) is similar as in previously published cohorts, i.e. between 11 and 22% [2, 5, 6, 14, 15]. We found even higher (30.3%) frequency of *TTN* trunc variants in familial DCM again similar to previous reports (between 18 and 27%). Our young DCM cohort was characterized by advanced heart failure with nearly half of the patients with documented familial disease. This is in agreement with current guidelines recommending genetic testing mainly in patients with clear-cut phenotype. As in the study by Herman et al. [2], subjects with DCM, both with and without *TTN* truncating variants, had generally similar clinical manifestations including the risk of major cardiac events. Similarly, as in the study by Herman et al., we found that in our patients DCM caused by *TTN* trunc is usually unaccompanied by conduction disease (in particular none of the *TTN* trunc carriers had AVB).

In our group 82.2% *TTN* truncating mutations were located in the symmetric exons of an A-band region. The A-band region, critical for biomechanical sensing and signaling, is organized in repeats containing numerous FN-III domains interspersed with Ig domains providing repetitive binding sites for myosin and thick filament associated proteins [16]. Our findings are consistent with the recent study by Roberts et al. who demonstrated that in end-stage DCM *TTN* truncating mutations occur predominantly in the A-band [6].

Only one proband had *TTN* truncating variant in the Z-disc region which confirms that this region is rarely mutated in patients with DCM. To our best knowledge only a few *TTN* variants located in Z-disc have been published so far in patients with DCM. These include three missense mutations [14, 17] and two truncating variants [6]. As penetrance was nearly full in our family with Z-disc variant we did not exclude the family from survival analyses.

*TTN* truncating mutations are more likely to be disease-causing when they occur in exons which are abundantly transcribed and located closer to the carboxyl end of the protein [6]. Our results suggest that *TTN* truncating variants, even when they meet the criteria mentioned above, are characterized by incomplete and sex-related penetrance. We found incomplete penetrance in *TTN* trunc relatives carriers that was 62.1% when all affected statuses were combined and 82% after exclusion of unaffected relatives under 40. In the study by Akinrinade et al. disease penetrance increased from 53.8% at age 50 to 84.6% at age 60 and 100% at age 70 [3]. In the study by Jansweijer et al, the penetrance of DCM in *TTN* relatives was 29% at the age of 50 (n = 49), had increased to 58% at the age of 60 (n = 43), and further to 83% at the age of 70 (n = 36) [18]. The varied and incomplete penetrance suggests a potential role of other genetic/environmental factors in the disease onset in DCM related titinopathy. It was shown by Cheng et al. that levels of genes’ expression can vary even among individuals of the same family and in this way influence the penetrance of mutation [19]. Furthermore, manifestation of clinical symptoms of pathogenic mutations is more likely to appear with increasing age of individual which, again, was showed for HCM and *MYBPC3* mutations [20] or Emery-Dreifuss muscular dystrophy and *LMNA* [21]. Our data show for the first time strong sex related difference with regard to estimated median age of disease onset in men (median 28 years) and women (median 56 years, p = 0.004). Furthermore, we found better prognosis (lower incidence of serious adverse cardiac events) in women than in men carrying *TTN* truncating mutations which is similar as in *LMNA* mutation carriers [22], and consistent with the report of Herman et al. [2]. The sex related difference in disease severity is interesting as it suggests a potential for interventions ameliorating DCM course in males. In particular, it would be interesting to study whether the higher disease severity in males could be related to the recently demonstrated link between TTN phosphorylation and increased oxidative stress [23].

Of note, severity of the disease and rates of major adverse cardiac events defined as cardiac transplantation, implantation of a left ventricular assist device and death from cardiac causes, were not different when comparing DCM probands and relatives affected with DCM.

## Limitations

Our population drawn from patients referred for clinical genetic testing introduces some bias towards more severely ill, with prominent family history. Also our Institute is one of leading cardiac transplant centers in Poland, patients with more advanced diseases are referred here, and patients with familial background are more willing to have their disease thoroughly clarified. Furthermore, we acknowledge that our findings should be interpreted with caution due to small patient numbers.

During a long period of time when probands were tested NGS procedures were improved and refined which led to wide ranges of NGS parameters like ge10 or sequencing depth. The minimum coverage of sequencing was showed as mean ge10 instead of widely used mean ge20 because this parameter was more stable than ge20.

## Conclusions

*TTN* truncating variants were observed in nearly one fourth of young DCM probands with sex-related differences in disease penetrance and severity which should be taken into account in genetic counselling.

## Supporting Information

S1 Fig Pedigrees of first 9 of 17 families with *TTN* truncating mutations. Squares represent males and circles represent females. An arrowhead denotes the proband. A diagonal line marks deceased individuals. Solid symbols denote affected status, half blackened symbols denote probably affected, quarter blackened symbols denote possibly affected status, open symbols with “N” denote not-affected individuals. Grey symbols denote individuals treated for heart failure but not tested at the reference center. The presence or absence of *TTN* mutation is indicated by a + or − symbol respectively. The presence or absence of additional variants are noted in parenthesis (+) or (-) respectively. DCM008: *TTN* p.Arg31056*, DCM019: *TTN* p.Arg21009*, DCM023: *TTN* p.Gly18918Valfs*17 (and *TNNI3* p.His34Gln), DCM029: *TTN* p.Gln27004*, DCM033: *TTN* p.Ile26829Metfs*15, DCM036: *TTN* p.Lys27131*, DCM075: *TTN* p.Arg22817*, DCM078: *TTN* p.Lys14528* (and *LDB3* p.Gly19Ala and *SCN5A* p.Ala572Asp), DCM081: *TTN* p. Ser28693Ilefs*2 (and *MYH6* p.Arg204His).
(TIF)

S2 Fig Pedigrees of remaining 8 of 17 families with *TTN* truncating mutations. For Fig legend see S1 Fig. DCM082: *TTN* p.Ala29119Leufs*17, DCM092: *TTN* p.Ser29255Alafs*18, DCM097: *TTN* p.Ser493* (and *DSP* p.Ala566Thr), DCM102: *TTN* p.Ala29119Leufs*17, DCM109: *TTN* p.Asn30734Glnfs*17 (and *PKP2* p.Pro7Ser), DCM113: *TTN* p.Arg17736* (and *ACTN2* p.Arg298His), DCM132: *TTN* p.Glu23514*, DCM134: *TTN* p.Gln26147* (and *MYH7* p.Arg237Trp).
(TIF)

S1 Table The NGS approach applied to probands with *TTN* truncating variants. (DOC)

S2 Table List of primers specific to *TTN* truncating variants found in this study. (DOC)

S3 Table Molecular characteristics of *TTN* truncating variants in our study. (DOC)

S4 Table List of missense variants found in our study group. Variants annotated to NM_001267550.2 transcript.
(DOCX)

S5 Table Comparison of *TTN* trunc positive DCM probands with *TTN* trunc positive DCM relatives. For Table Legend see Table 2.
(DOC)

S6 Table Bioinformatics predictions of pathogenicity of additional variants found in probands carrying TTN truncating mutations. Legend: PolyPhen2: D-probably damaging, P-possibly damaging, B-benign; MutationTaster: D-disease casing, N-polymorphism.
(DOCX)
