# Supplementary figures and images for: Titin Truncating Variants in Dilated Cardiomyopathy – Prevalence and Genotype-Phenotype Correlations
Source: PLoS One. 2017 Jan 3;12(1):e0169007. doi: 10.1371/journal.pone.0169007 (PMC5207678; doi:10.1371/journal.pone.0169007)

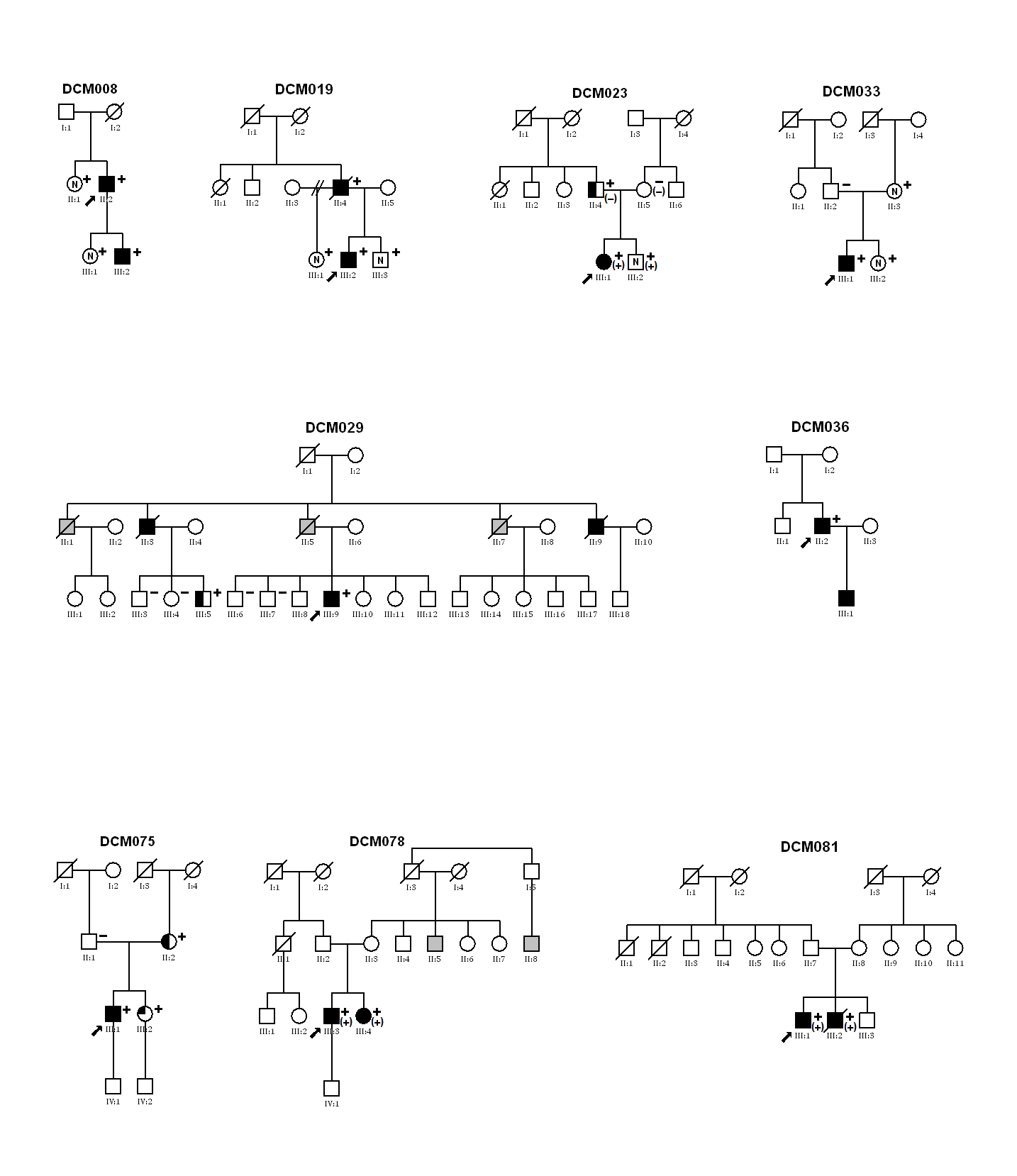

Supplement: S1 Fig — Squares represent males and circles represent females. An arrowhead denotes the proband. A diagonal line marks deceased individuals. Solid symbols denote affected status, half blackened symbols denote probably affected, quarter blackened symbols denote possibly affected status, open symbols with “N” denote not-affected individuals. Grey symbols denote individuals treated for heart failure but not tested at the reference center. The presence or absence of TTN mutation is indicated by a + or − symbol respectively. The presence or absence of additional variants are noted in parenthesis (+) or (-) respectively. DCM008: TTN p.Arg31056*, DCM019: TTN p.Arg21009*, DCM023: TTN p.Gly18918Valfs*17 (and TNNI3 p.His34Gln), DCM029: TTN p.Gln27004*, DCM033: TTN p.Ile26829Metfs*15, DCM036: TTN p.Lys27131*, DCM075: TTN p.Arg22817*, DCM078: TTN p.Lys14528* (and LDB3 p.Gly19Ala and SCN5A p.Ala572Asp), DCM081: TTN p. Ser28693Ilefs*2 (and MYH6 p.Arg204His). (TIF) [file pone.0169007.s001.tif]

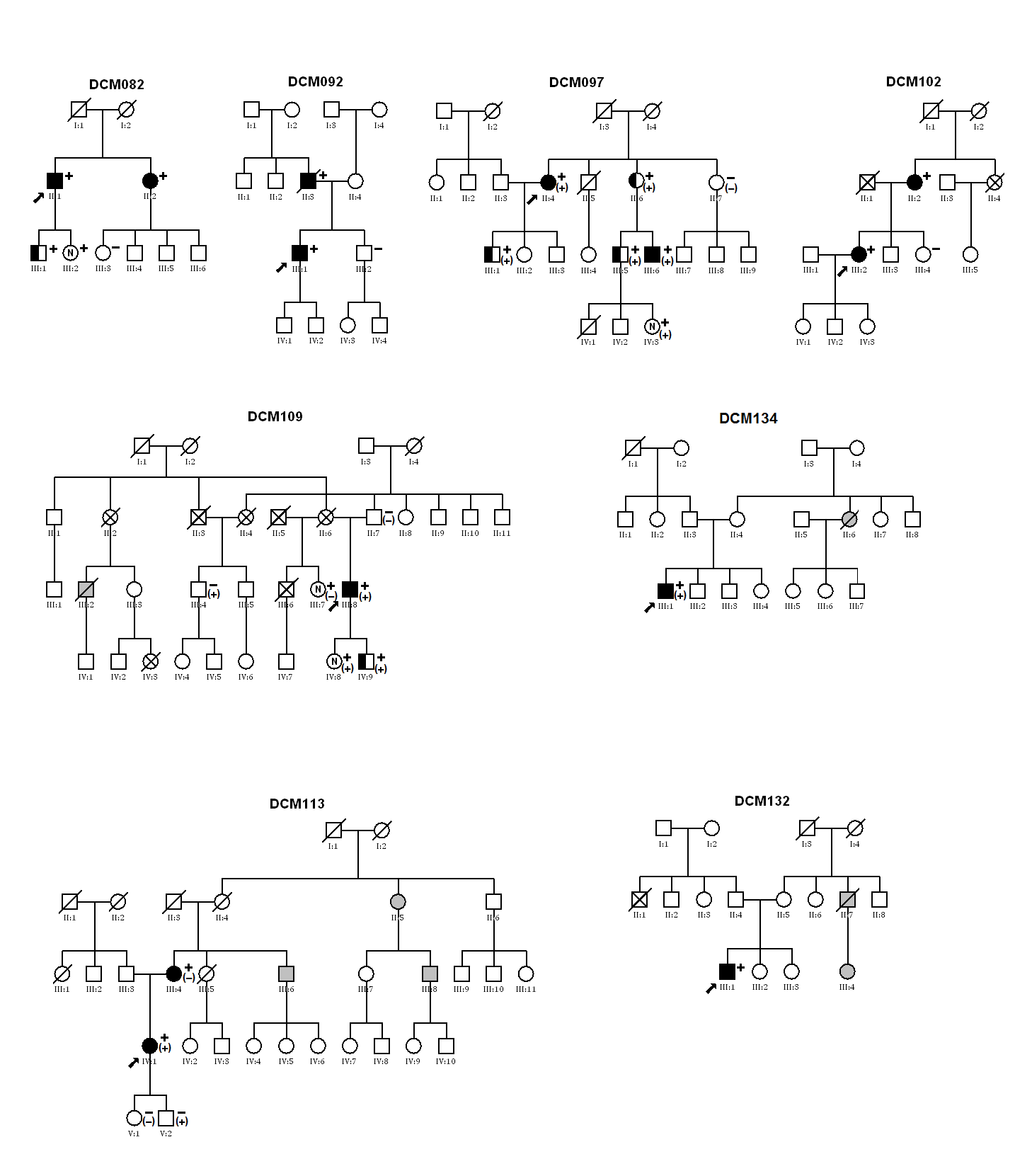

Supplement: S2 Fig — For Fig legend see S1 Fig. DCM082: TTN p.Ala29119Leufs*17, DCM092: TTN p.Ser29255Alafs*18, DCM097: TTN p.Ser493* (and DSP p.Ala566Thr), DCM102: TTN p.Ala29119Leufs*17, DCM109: TTN p.Asn30734Glnfs*17 (and PKP2 p.Pro7Ser), DCM113: TTN p.Arg17736* (and ACTN2 p.Arg298His), DCM132: TTN p.Glu23514*, DCM134: TTN p.Gln26147* (and MYH7 p.Arg237Trp). (TIF) [file pone.0169007.s002.tif]
