# Supplementary material for: Titin Truncating Variants in Dilated Cardiomyopathy – Prevalence and Genotype-Phenotype Correlations
Source: PLoS One. 2017 Jan 3;12(1):e0169007. doi: 10.1371/journal.pone.0169007 (PMC5207678; doi:10.1371/journal.pone.0169007)
Supplement: S1 Table — (DOC) [file pone.0169007.s003.doc]

**S1 Table. The NGS approach applied to probands with *TTN* truncating variants**

| **Family** | **NGS approach** |
| --- | --- |
| DCM008 | WES |
| DCM019 | WES |
| DCM023 | WES |
| DCM029 | WES |
| DCM033 | WES |
| DCM036 | TSO |
| DCM075 | WES |
| DCM078 | WES |
| DCM081 | WES |
| DCM082 | WES |
| DCM092 | WES |
| DCM097 | WES |
| DCM102 | WES |
| DCM109 | WES |
| DCM113 | TSO |
| DCM132 | WES |
| DCM134 | TSO |
