# Supplementary material for: Titin Truncating Variants in Dilated Cardiomyopathy – Prevalence and Genotype-Phenotype Correlations
Source: PLoS One. 2017 Jan 3;12(1):e0169007. doi: 10.1371/journal.pone.0169007 (PMC5207678; doi:10.1371/journal.pone.0169007)
Supplement: S2 Table — (DOC) [file pone.0169007.s004.doc]

**S2 Table. List of primers specific to *TTN* truncating variants found in this study**

| ***TTN* truncating variant** | **FORWARD** | **REVERSE** |
| --- | --- | --- |
| p.Ser493*/c.1478C>A | GCGAGGCTGGTCTTGAACTA | CAACACTCTTCATGGTAAAGGTGA |
| p.Lys14528*/c.43582A>T | GGCAAACTGATCATTGAAGG | TTTCATGCAATATAACACTTAGAAGAC |
| p.Arg17736*/c.53206C>T | TGGCTGAACCACAAGGTACT | TGGACCAGGGACATCTGAA |
| p.Gly18918Valfs*17/c.56751_56752delAG | GACAGTGAACCTGAAACAGCA | AGGCACTTTTGGAAAATAAGATT |
| p.Arg21009*/c.63025C>T | ATAAACCTGGTCGCCCTGA | AAGGCAAGCTTGGTTCTCC |
| p.Arg22817*/c.68449C>T | TGTATAAATAATTTGGGCAACACA | CCTCAGGTTTTCCAGGAGGA |
| p.Glu23514*/c.70540G>T | CATCACAAAGGACAGTGTCACC | CCACTGGTCAGAGCCTTTTC |
| p.Gln26147*/c.78439C>T | GATCCCTGTGACCCACCA | CCATGGACATCAGCCTCAAG |
| p.Ile26829Metfs*15/c.80486delT | CCAGTGGAAACTGTTGATGC | TGACAGGAACACCCAACACT |
| p.Gln27004*/c.81010C>T | GGCACAGCAACAGAAAATCTC | GGGGCACTTTTTCCATACCT |
| p.Lys27131*/c.81391A>T | CCATTTAAAGAACCTGGACCAC | GTAAAGCTGGCTTTCATCCA |
| p.Ser28693Ilefs*2/c.86078insA | CAAGACCCGAGAGTGATGGA | ATCACTGGGGTCACTAGCAC |
| p.Ala29119Leufs*17/c.87355delG | CTGGAAAACCACTTCCCAAA | AGCGGTTTTCTGCCTTGAT |
| p.Ser29255Alafs*18/c.87757delA | AGGTTTTCTTTTACCATAAATGTGTT | GCAGAAGTAAGATTTGCATTTTTG |
| p.Asn29093Glnfs*17/c.87277insC | GCAAAAGCAGAAATTAAAGTGAAA | TTGGTCGTTTGCTGATCACT |
| p.Arg29415*/c.88243C>T | AGATTCCTTCAGCACCCTCA | AGGGCTCACCAACACCATAC |
