# Supplementary material for: Titin Truncating Variants in Dilated Cardiomyopathy – Prevalence and Genotype-Phenotype Correlations
Source: PLoS One. 2017 Jan 3;12(1):e0169007. doi: 10.1371/journal.pone.0169007 (PMC5207678; doi:10.1371/journal.pone.0169007)
Supplement: S3 Table — (DOC) [file pone.0169007.s005.doc]

**S3 Table. Molecular characteristics of *TTN*** truncating variants in our study

| **Family** | **Genomic position** | **Variant** | **Transcripts** | **Exon** | **Protein region of sarcomere** | **S/A** | **Average exon PSI (DCM)** | **Average exon PSI (GTEx)** |
| --- | --- | --- | --- | --- | --- | --- | --- | --- |
| DCM008 | chr2:179413187 | NM_001256850.1:p.Arg29415*/c.88243C>T,  NM_001267550.2:p.Arg31056*/c.93166C>T | N2A, N2B, N2AB, novex 1 i 2 | 340 | A-band | S | 1.00 | 0.98 |
| DCM019 | chr2:179453427 | NM_001256850.1:p.Arg19368*/c.58102C>T,  NM_001267550.2:p.Arg21009*/c.63025C>T | N2A, N2B, N2AB, novex 1 i 2 | 305 | A-band | S | 1.00 | 0.97 |
| DCM023 | chr2:179463684 | NM_001256850.1:p.Gly17277Valfs*17/c.51828_51829delAG,  NM_001267550.2:p.Gly18918Valfs*17/c.56751_56752delAG | N2A, N2B, N2AB, novex 1 i 2 | 292 | A-band | S | 1.00 | 0.94 |
| DCM029 | chr2:179429849 | NM_001256850.1:p.Gln25363*/c.76087C>T,  NM_001267550.2:p.Gln27004*/c.81010C>T | N2A, N2B, N2AB, novex 1 i 2 | 327 | A-band | S | 1.00 | 0.95 |
| DCM033 | chr2:179430371 | NM_001256850.1:p.Ile25188Metfs*15/c.75564delT,  NM_001267550.2:p.Ile26829Metfs*15/c.80487delT | N2A, N2B, N2AB, novex 1 i 2 | 327 | A-band | S | 1.00 | 0.95 |
| DCM036 | chr2:179429468 | NM_001256850.1:p.Lys25490*/c.76468A>T,  NM_001267550.2:p.Lys27131*/c.81391A>T | N2A, N2B, N2AB, novex 1 i 2 | 327 | A-band | S | 1.00 | 0.95 |
| DCM075 | chr2:179442793 | NM_001256850.1:p.Arg21176*/c.63526C>T,  NM_001267550.2:p.Arg22817*/c.68449C>T | N2A, N2B, N2AB, novex 1 i 2 | 323 | A-band | S | 1.00 | 0.91 |
| DCM078 | chr2:179497039 | NM_001256850.1:p.Lys12887*/c.38659A>T,  NM_001267550.2:p.Lys14528*/c.43582A>T | N2A, N2B, N2AB, novex 1 i 2 | 237 | I-band | S | 1.00 | 0.98 |
| DCM081 | chr2:179424782 | NM_001256850.1:p.Ser27052Ilefs*2/c.81153_81154insA,  NM_001267550.2:p.Ser28693Ilefs*2/c.86076_86077insA | N2A, N2B, N2AB, novex 1 i 2 | 327 | A-band | S | 1.00 | 0.95 |
| DCM082 | chr2:179422725 | NM_001256850.1:p.Ala27478Leufs*17/c.82432delG,  NM_001267550.2:p.Ala29119Leufs*17/c.87355delG | N2A, N2B, N2AB, novex 1 i 2 | 329 | A-band | S | 1.00 | 0.95 |
| DCM092 | chr2:179422231 | NM_001256850.1:p.Glu27613Alafs*18/c.82834delA,  NM_001267550.2:p.Glu29254Alafs*18/c.87757delA | N2A, N2B, N2AB, novex 1 i 2 | 330 | A-band | S | 1.00 | 0.96 |
| DCM097 | chr2:179658189 | NM_001256850.1:p.Ser493*/c.1478C>A,  NM_001267550.2:p.Ser493*/c.1478C>A | N2A, N2B, N2AB, novex 1 i 2 | 9 | Z-disc | S | 1.00 | 1.00 |
| DCM102 | chr2:179422725 | NM_001256850.1:p.Ala27478Leufs*17/c.82432delG,  NM_001267550.2:p.Ala29119Leufs*17/c.87355delG | N2A, N2B, N2AB, novex 1 i 2 | 329 | A-band | S | 1.00 | 0.95 |
| DCM109 | chr2:179414153 | NM_001256850.1:p.Asn29093Glnfs*17/c.87276_87277insC,  NM_001267550.2:p.Asn30734Glnfs*17/c.92199_92200insC | N2A, N2B, N2AB, novex 1 i 2 | 340 | A-band | S | 1.00 | 0.98 |
| DCM113 | chr2:179472209 | NM_001256850.1:p.Arg16095*/c.48283C>T,  NM_001267550.2:p.Arg17736*/c.53206C>T | N2A, N2B, N2AB, novex 1 i 2 | 278 | A-band | S | 1.00 | 0.94 |
| DCM132 | chr2:179440319 | NM_001256850.1:p.Glu21873*/c.65617G>T,  NM_001267550.2:p.Glu23514*/c.70540G>T | N2A, N2B, N2AB, novex 1 i 2 | 327 | A-band | S | 1.00 | 0.95 |
| DCM134 | chr2:179432420 | NM_001256850.1:p.Gln24506*/c.73516C>T,  NM_001267550.2:p.Gln26147*/c.78439C>T | N2A, N2B, N2AB, novex 1 i 2 | 327 | A-band | S | 1.00 | 0.95 |
| Variant - Variant in transcript NM_001267550; Transcripts - *TTN* transcripts containing variant; Exon - Exon number in NM_001267550 transcript, S/A - symmetric or asymetric exon, PSI - Percent Splicing Index based on Roberts et al. | | | | | | | | |
