# Supplementary material for: Titin Truncating Variants in Dilated Cardiomyopathy – Prevalence and Genotype-Phenotype Correlations
Source: PLoS One. 2017 Jan 3;12(1):e0169007. doi: 10.1371/journal.pone.0169007 (PMC5207678; doi:10.1371/journal.pone.0169007)
Supplement: S4 Table — Variants annotated to NM_001267550.2 transcript. (DOCX) [file pone.0169007.s006.docx]

**S4 Table. List of missense variants found in our study group.** Variants annotated to NM_001267550.2 transript

| **Genomic position** | ***TTN* missense variants** | **Proband with TTN trunc** |
| --- | --- | --- |
| chr2:179544633 | p.Ile11190Val/c.33568A>G | no |
| chr2:179593059 | p.Ile6498Leu/c.19492A>C | yes |
| chr2:179427578 | p.Val27761Leu/c.83281G>C | yes |
| chr2:179474438 | p.Pro17238Ser/c.51712C>T | yes |
| chr2:179431967 | p.Gly26298Arg/c.78892G>A | yes |
| chr2:179396214 | p.Arg35043His/c.105128G>A | yes |
| chr2:179418306 | p.Arg29809Gln/c.89426G>A | yes |
| chr2:179425988 | p.Arg28291Cys/c.84871C>T | yes |
| chr2:179458059 | p.Ala19626Thr/c.58876G>A | yes |
| chr2:179449452 | p.Arg21639Gln/c.64916G>A | no |
| chr2:179595389 | p.Gln5957His/c.17871A>T | no |
| chr2:179550046 | p.Gln10802Lys/c.32404C>A | no |
| chr2:179452447 | p.Ile21197Val/c.63589A>G | no |
| chr2:179429404 | p.Ile27152Thr/c.81455T>C | no |
| chr2:179422714 | p.Ser29123Arg/c.87367A>C | no |
| chr2:179411522 | p.Arg31545Cys/c.94633C>T | 3*no |
| chr2:179605212 | p.Val4250Met/c.12748G>A | 3*no |
| chr2:179596554 | p.Tyr5683Cys/c.17048A>G | no |
| chr2:179500821 | p.Ile13826Thr/c.41477T>C | no |
| chr2:179501304 | p.Thr13717Ile/c.41150C>T | no |
| chr2:179474228 | p.Ser17270Ile/c.51809G>T | no |
| chr2:179419353 | p.Arg29574His/c.88721G>A | no |
| chr2:179435906 | p.Ala24985Ser/c.74953G>T | no |
| chr2:179412986 | p.Val31123Ile/c.93367G>A | no |
| chr2:179605815 | p.Pro4049Ser/c.12145C>T | yes |
| chr2:179596269 | p.Leu5742Phe/c.17224C>T | no |
| chr2:179397654 | p.Val34563Ala/c.103688T>C | no |
| chr2:179401935 | p.Glu33301Lys/c.99901G>A | no |
| chr2:179436668 | p.Glu24731Lys/c.74191G>A | yes |
| chr2:179559353 | p.Val10467Ile/c.31399G>A | 1*yes, 1*no |
| chr2:179539777 | p.Leu11534Pro/c.34601T>C | no |
| chr2:179606403 | p.Trp3853Arg/c.11557T>A | no |
| chr2:179446227 | p.Leu22256Phe/c.66768A>C | no |
| chr2:179393658 | p.Ala35607Val/c.106820C>T | no |
| chr2:179658229 | p.Val480Leu/c.1438G>C | no |
| chr2:179425177 | p.Ser28561Cys/c.85682C>G | no |
| chr2:179574418 | p.Thr9543Ile/c.28628C>T | no |
| chr2:179638579 | p.Arg2439His/c.7316G>A | no |
| chr2:179392461 | p.Pro35798Thr/c.107392C>A | no |
| chr2:179417400 | p.Thr30076Met/c.90227C>T | no |
| chr2:179647706 | p.Trp976Leu/c.2927G>T | no |
