# Supplementary material for: Titin Truncating Variants in Dilated Cardiomyopathy – Prevalence and Genotype-Phenotype Correlations
Source: PLoS One. 2017 Jan 3;12(1):e0169007. doi: 10.1371/journal.pone.0169007 (PMC5207678; doi:10.1371/journal.pone.0169007)
Supplement: S5 Table — For Table Legend see Table 2. (DOC) [file pone.0169007.s007.doc]

**S6 Table. Comparison of *TTN* trunc positive DCM probands with *TTN* trunc positive definitely affected DCM relatives**

|  | **All *TTN* trunc (+) carriers with DCM** | ***TTN* trunc (+) probands** | ***TTN* trunc (+) affected relatives** | **p** |
| --- | --- | --- | --- | --- |
| **N** | 26 | 17 | 9 |  |
| **Age at diagnosis** | 36.3±14.0 | 33.3±11.1 | 41.8±17.7 | 0.16 |
| **Male sex n (%)** | 65.4% (16) | 70.6% (12) | 55.6% (5) | 0.39 |
| **Symptoms** | | | | |
| **Acute onset heart failure** | 23.1% (6) | 23.5% (4) | 22.2% (2) | 1.00 |
| **Sudden cardiac arrest** | 3.8% (1) | 0 | 11.1% (1) | 0.32 |
| **Decreased exercise tolerance** | 69.2% (18) | 76.5% (13) | 55.6% (5) | 1.00 |
| **NYHA class at onset** | 2.9±0.8 | 2.9±0.8 | 3.0±0.9 | 0.90 |
| **Asymptomatic** | 3.8% (1) | 0 | 11.1% (1) |  |
| **Cardiac assessment** | | | | |
| **LVEF % (mean±SD)** | 25.8±10.1 | 24.5±9.0 | 28.1±12.1 | 0.82 |
| **Sinus rhythm** | 84.6% (22) | 88.2% (15) | 77.8% (7) | 0.57 |
| **AF/PAF** | 38.5% (10) | 35.3% (6) | 44.4% (4) | 1.00 |
| **LBBB and/or AVB** | 15.4% (4) | 5.9% (1) | 33.3% (3) | 0.23 |
| **Outcome** | | | | |
| **Time from diagnosis (months)** | 65.0 ±69.0 | 63.6±63.9 | 68±83.7 | 1.00 |
| **Major adverse cardiac events** | 30.8% (8) | 29.4% (5) | 33.3% (3) | 1.00 |
| **HF Death** | 7.7% (2) | 0 | 22.2% (2) | 0.09 |
| **HTX** | 19.2% (5) | 23.5% (4) | 11.1% (1) | 1.00 |
| **LVAD as bridge to recovery** | 3.7% (1) | 5.9% (1) | 0 | 1.00 |
