# Supplementary material for: Titin Truncating Variants in Dilated Cardiomyopathy – Prevalence and Genotype-Phenotype Correlations
Source: PLoS One. 2017 Jan 3;12(1):e0169007. doi: 10.1371/journal.pone.0169007 (PMC5207678; doi:10.1371/journal.pone.0169007)
Supplement: S6 Table — Legend: PolyPhen2: D-probably damaging, P-possibly damaging, B-benign; MutationTaster: D-disease casing, N-polymorphism. (DOCX) [file pone.0169007.s008.docx]

**S7 Table.** Bioinformatics predictions of pathogenicity of additional variants found in probands carrying TTN truncating mutations.

| **Family** | **Gene** | **Transcript** | **Variant** | **AVSIFT** | **PolyPhen2** | **Mutation**  **Taster** | **ClinVar** |
| --- | --- | --- | --- | --- | --- | --- | --- |
| DCM078 | LDB3 | NM_001171610.1 | p.Gly19Ala | 0.00 | P (0.789) | D | No data |
| DCM078 | SCN5A | NM_000335 | p.Ala572Asp | 0.22 | B (0.055) | N | Benign/likely benign |
| DCM023 | TNNI3 | NM_000363.4 | p.His34Gln | 0.31 | B (0.031) | N | No data |
| DCM109 | PKP2 | NM_001005242.2 | p.Pro7Ser | 0.51 | B (0.000) | N | VUS |
| DCM097 | DSP | NM_001008844 | p.Ala566Thr | 0.05 | P (0.778) | D | VUS |
| DCM113 | ACTN2 | NM_001103.3 | p.Arg298His | 0.00 | B (0.001) | D | VUS |
| DCM134 | MYH7 | NM_000257.3 | p.Arg237Trp | 0.00 | D (1.000) | D | Pathogenic/likely pathogenic/VUS |
| DCM081 | MYH6 | NM_002471.3 | p.Arg204His | 0.01 | P (0.346) | D | No data |
| Legend: PolyPhen2: D-probably damaging, P-possibly damaging, B-benign; MutationTaster: D-disease casing, N-polymorphism, VUS-variant of unknown significance. | | | | | | | |
